# Prevalence of human immunodeficiency virus and hepatitis B virus infections in young women seeking abortion care in Ethiopia: a cross - sectional study

**DOI:** 10.1186/s12889-016-3658-9

**Published:** 2016-09-20

**Authors:** Wondemagegn Mulu, Yohannes Zenebe, Bayeh Abera, Mulat Yimer, Tadesse Hailu

**Affiliations:** Department of Medical Microbiology, Immunology and Parasitology, College of Medicine and Health Sciences, Bahir Dar University, Bahir Dar, Ethiopia

**Keywords:** HIV, HBV, Young women, Abortion care

## Abstract

**Background:**

Young women aged 15–24 years are members of key populations at higher risk for Human Immunodeficiency Virus (HIV) acquisition through sexual intercourse. In areas where unprotected sex is a common practice, Hepatitis B virus (HBV) commonly transmitted via sexual and parenteral routes. The study aimed at determining HIV and HBV infections prevalence in young women attending health institutions for abortion care in Bahir Dar city, Ethiopia.

**Methods:**

A cross - sectional study was conducted from January 2015 to June 2015. Convenient sampling technique was used. Demographic and explanatory variables were collected using a structured questionnaire via face to face interview. The presence of antibody to HIV infection was detected using national HIV diagnostic test algorithm. Hepatitis B surface antigen (HBsAg) was detected using ELISA. Data were analyzed using descriptive, fisher’s exact and independent sample *T* test as appropriate.

**Results:**

A total of 360 young women aged 15–24 years participated in the study. The median age of the women was 22 years. Overall, 16 (4.4 %) (95 % CI: 2.7–7.1 %) women were positive for either HBV or HIV infections. The prevalence of HIV and HBV infections were 9 (2.5 %) (95 % CI: 1.3–4.7 %) and 7 (1.94 %) (95 % CI: 0.95–4.0 %), respectively. The mean age of first sexual intercourse was 17.6 and 19.3 in HIV and HBV infected women, respectively. The prevalence of HIV infection was significantly associated with lower educational status (*P* < 0.001), divorced marital status (*P* = 0.009) and ever had symptom of other sexually transmitted infections (*P* = 0.001). The proportion of HBV was higher in women aged 15–17 years (*P* = 0.02).

**Conclusion:**

Though there were no co-infections, HIV and HBV infections are major health problems in young women seeking abortion care. Therefore, appropriate prevention, treatment and care services must be reached to these higher risk populations.

## Background

Women in sexual relationships and victims of sexual assault are at greater risk of getting Human Immunodeficiency Virus (HIV) and Hepatitis B virus (HBV) because of unsafe sexual practices [1, 2]. Presence of other sexually transmitted infections (STIs) increase the probability of HIV and HBV acquisition [1–3].

Contraceptive use remains very low in all regions of sub-Saharan Africa among women in 15 to 24 years [4]. Unwanted pregnancy is a current problem in Ethiopia [1, 4]. Sexually active women often face obstacles to access contraceptives and other health services, therefore predispose to unintended pregnancy [4]. Condom use as a birth control method is low in Ethiopia. Few adolescents take advantage of HIV testing and counseling services [1]. Studies stated that the proportion of women engaged in sexual activities before marriage increased and the age at first sexual intercourse decreased [1, 4]. These factors increase STIs among young women [1]. Abortion is one of the methods used for women who have an unwanted pregnancy for whatever reason [1]. High abortion rate and low contraceptive prevalence rates are common in women aged from 15 to 24 years in Ethiopia [4].

HIV and HBV infections are the two most important infectious diseases in developing countries [5]. They are transmitted through sexual contact, exposure to infected body fluids and from mother to fetus or child during prenatal period. Of these, sexual contact is the predominant mode of transmission [5, 6]. Transmission of HBV in sub-Saharan Africa commonly occurs during childhood. Sexual and parenteral routes are also important particularly in areas where unprotected sex is a common practice [7].

Globally, young people aged from 15 to 24 years continue to be vulnerable to HIV infection. Women in this age group are members of key populations at higher risk for HIV acquisition or transmission through sexual route [7–9]. Sub-Saharan Africa is a region with the highest numbers of HIV positive adolescents [10–12]. In 2012, the world HIV prevalence in youth aged 15–24 was 0.8 and 4.7 % in sub-Saharan Africa [7]. In sub-Saharan Africa women face higher risk of HIV acquisition in early ages because of biological factors and gender inequalities leading to unequal power relationships, access to education and economic opportunities [7].

Hepatitis B virus (HBV) infection is one of the leading causes of liver diseases causing serious public health problem worldwide [5, 13, 14]. One - third of the world’s populations have serologic evidence of HBV infection [11, 14]. Sub-Saharan Africa is considered a highly endemic area for HBV by WHO [7]. Among young women, the prevalence of HBV infection is 6–20 % and varies among different regions [11–13]. In Ethiopia, a 1.8–6.6 % and 3–7.3 % prevalence of HIV and HBV infections, respectively reported in other risk populations [15–18].

Due to very low rate of contraceptive use and coverage of hepatitis B vaccine, young women 15–24 years are the most vulnerable population in Ethiopia. However, there was no previous study conducted on HIV and HBV infections among such groups seeking abortion for unwanted pregnancies in Ethiopia. Therefore, determining the prevalence of HIV and HBV in these vulnerable populations helps to quantify the magnitude of STI risk and inform prevention effort. Moreover, reducing new HIV and HBV infections among women of reproductive age is important to keep women healthy, eliminate mother to child transmission and reduce the burden of HIV and HBV in the community. This study was conducted to determine the prevalence of HBV and HIV infections with associated factors among women aged from 15 to 24 years seeking abortion care at Bahir Dar city, Ethiopia.

## Methods

### Study design

A cross - sectional study was conducted from January 2015 to June 2015 at the Family Guidance Association (FGA) and Marie Stopes International Ethiopia, Bahir Dar city. Bahir Dar city is the capital of Amhara National Regional State having a total population of 311,724. According to Ethiopia Mini Demographic and Health Survey 2014 report, the contraceptive rate was 9.3 and 31.9 % for women aged 15–19 and 20–24 years, respectively [19].

Family Guidance Association and Marie Stopes International Ethiopia clinics provide different services for young youths and adolescents. They provide sexual reproductive health, family planning, adolescent and youth, comprehensive abortion care, STI prevention, safe mother hood initiative (prenatal, natal and postnatal), immunization and medical services. The daily work load of the two clinics is 80–85 women to deliver the above mentioned services. In each clinic, 3–5 young women attend daily for comprehensive abortion care.

### Sample size and sampling

The study population was all young women in reproductive age (15–24 years) attending FGA and Marie Stopes International Ethiopia, Bahir Dar, for abortion of unwanted pregnancies. The sample size was determined using single population proportion formula (N = z2 p (1-p)/d2) [20], considering 95 % confidence level, 0.5 proportion of HIV or HBV infection, marginal error of (5 %). Thus, a sample size of 384 was calculated. Convenient sampling technique was used to enroll the study participants. All young women aged 15–24 years attending the two clinics and volunteered to participate included until the sample size completed.

### Inclusion criteria

All young women aged 15–24 years attending FGA and Marie Stopes International Ethiopia clinics for safe abortion of unwanted pregnancies were included.

### Exclusion criteria

All young women aged 15–24 years attending FGA and Marie Stopes International Ethiopia clinics that were seropositive either for HIV or HBV before were excluded from this study.

### Variables of the study

HIV and HBV infections were the dependent variables whereas demographic and other explanatory variables such as age, residence, religion, educational and marital status, occupation, history of abortion, blood transfusion, tooth extraction, history of genital mutilation, history of surgical operation, history of needle stick injury, sharing of sharp objects, type of sexual partner, alcohol drinking habit, ever had symptom of STI and mean age of first sexual intercourse were the independent variables.

### Data collection

Before the actual data collection, questionnaires were pre-tested by taking 36 young women aged 15–24 years attending FGA and Marie Stopes International Ethiopia, Bahir Dar clinics seeking comprehensive abortion care other than the actual study participants. Upon counseling and recruitment, information on demographic and explanatory variables was collected by face to face interview using a structured questionnaire. Interviewees conducted by trained medical doctors and clinical nurses working as HIV testing and counseling in the youth center of the clinics.

### Laboratory investigation

Five ml of whole blood through vein puncture was collected aseptically by trained laboratory technologist from study participants. Serum was separated by centrifugation for 5 min at room temperature and capped in nunck tube and stored at −20 °C. The presence of hepatitis B surface antigens (HBsAg) in serum was detected using ELISA according to the manufacturer’s instruction (DIALAB GmbH, Austria). Antibody to HIV infection was tested using the national HIV rapid diagnostic test algorithm. Initially, HIV infection was screened using KHB (Bio-Engineering, Shanghai, Kehua). HIV positive samples were re-tested with STAT PAK (Chembio diagnostic, INC, Medfold, Newyork). Samples yielding discordant results between the first and the second tests were tested again with Unigold (Trinity Biotech PLC, Bray, Ireland). The results were interpreted using the current national algorithm for screening of HIV infection from whole blood which was adopted from WHO [21].

### Quality control

Questionnaire was pretested on a smaller number of youth women other than the study participants. The collected data was checked daily for consistency and accuracy. Standardized procedures were strictly followed during blood sample collection, storage and analytical process. Positive and negative controls were run alongside of the test.

### Data analysis

Data were entered and analyzed using Statistical Package for Social Sciences (SPSS) version 20. Descriptive statistics were used to describe the study participants in relation to relevant variables. Fisher’s exact tests were used to compare the distribution of all demographic and explanatory variables between HIV and HBV positive and negative participants. Independent sample T tests were used to compare the distribution of mean age of first sexual intercourse and mean number of sexual partners for the last one year between HIV and HBV positive and negative participants. *P*-value of < 0.05 was considered statistical significant.

## Results

### Demographic characteristics

A total of 360 young women with 93.8 % response rate participated in the study. Of whom, 293 (67.3 %) were living in an urban setting. The median age of the participants was 22 years. Majority (36.9 %) of participants were employees. In terms of educational levels, 153 (42.5 %) were Grade 9–12. The majority were single (81.1 %) and orthodox Christian followers (92.5 %) (Table 1). Twenty (5.6 %) of participants ever had symptom of sexually transmitted infections (Table 2).

**Table 1:** Prevalence of HBV and HIV infections among young women aged from 15 to 24 years seeking abortion care at Bahir Dar city, 2015 (*n* = 360)

Demographic variables	HBsAg		*P*-value	HIV status		*P*-value
	Positive N (%)	Negative N (%)		Positive N (%)	Negative N (%)	
Residence						
Urban	6 (2)	287 (98)	1.00	8 (2.72)	286 (97.3)	0.49
Rural	1 (1.5)	65 (98.5)		1 (1.5)	65 (98.8)	
Age in years						
15–17	1 (16.7)	5 (83.3)	0.02	0	6 (100)	
18–20	1 (0.8)	119 (99.2)		1 (0.8)	120 (99.2)	
21–24	5 (2.1)	228 (97.9)		8 (3.4)	225 (96.6)	0.31
Religion						
Orthodox	7 (2.1)	326 (97.9)		9 (2.7)	325 (97.3)	0.69
Protestant	0	11 (100)		0	11 (100)	
Muslim	0	15 (100)		0	15 (100)	
Educational status						
Illiterate	0	33 (100)	0.12	4 (12.9)	29 (87.1)	<0.001
Grade 1–6	2 (10)	18 (90)		3 (15)	17 (85)	
Grade 7–8	0	33 (100)		0	33 (100)	
Grade 9–12	2 (1.3)	151 (98.7)		1 (0.7)	152 (99.3)	
University students	1 (2.1)	47 (97.9)		1 (2)	48 (98)	
Diploma & above	2 (2.8)	70 (97.2)		0	71 (100)	
Marital status						
Single	6 (2)	286 (98)	0.26	6 (2)	287 (98)	
Married	0	54 (100)		1 (1.9)	53 (98.1)	
Divorced	1 (10)	10 (90)		2 (18.2)	9 (81.8)	0.009
Other	0	2 (100)		0	2 (100)	
Occupation						
Students	2 (1.7)	116 (98.3)	0.67	1 (0.9)	117 (99.1)	
Employees	4 (3)	129 (97)		5 (3.8)	128 (96.2)	0.39
House wife	0	21 (100)		0	21 (100)	
Other	1 (1.1)	87 (98.9)		3 (3.4)	85 (96.6)	
Total	7 (1.94)	353 (98.1)		9 (2.5)	351 (97.5)

**Table 2:** Association of explanatory variables and HIV infection in young women aged from 15 to 24 years seeking abortion care at Bahir Dar city, 2013 (*n* = 360)

Variables	HIV status		*P*-value
	Positive N (%)	Negative N (%)	
History of blood transfusion			
Yes	0	5 (100)	
No	9 (2.5)	346 (97.5)	0.88
History of tooth extraction			
Yes	2 (4.7)	41 (95.3)	0.29
No	7 (2.2)	310 (97.8)	
History of genital mutilation			
Yes	5 (3.1)	155 (96.9)	0.36
No	4 (2)	196 (98)	
History of surgical operation			
Yes	1 (5)	19 (95)	0.41
No	8 (2.4)	332 (97.6)	
History of needle stick injury			
Yes	2 (1.94	101 (98.1)	0.49
No	7 (2.7)	250 (97.3)	
History of sharing of sharp objects			
Yes	1 (3.1)	31 (96.9)	0.57
No	8 (2.4)	320 (97.6)	
Unprotected sex with older man			
Yes	2 (4)	48 (98)	0.76
No	7 (2.3)	302 (97.7)	
Type of sexual partner			
Student	1 (1.4)	69 (98.6)	0.15
Business man	3 (4.3)	66 (95.7)	
Drivers	2 (4.2)	46 (95.8)	
Soldier/police man	0	10 (100)	
Teachers	0	17 (100)	
All types	1 (20)	4 (80)	
Other	2 (1.4)	139 (98.6)	
Alcohol drinking habit			
Yes	4 (9.5)	38 (90.5)	0.01
No	5 (1.6)	312 (98.4)	
Ever had symptom of STI			
Yes	4 (20)	16 (80)	0.001
No	5 (1.5)	335 (98.5)	
Mean age of first sexual intercourse	17.6	18.5	0.24
Mean number of sexual partners for the last 1 year	1.8	1.4	0.35

### Prevalence of HIV and HBV infection

Overall, 16 (4.4 %) (95 % CI: 2.7–7.1 %) of young women were positive either for HBsAg or HIV antibodies. The prevalences of HBsAg and HIV were 7 (1.94 %) (95 % CI: 0.95–4.0 %) and 9 (2.5 %) (95 % CI: 1.3–4.7 %), respectively. The prevalence of HIV infection was 4 (12.9 %) and 3 (15 %) among illiterates and grade 1–6, respectively (*P* < 0.001). It was higher in divorced 2 (18.2 %) women (*P* = 0.009) (Table 1). The mean age for first sexual intercourse in HIV infected women was 17.6 year. The prevalence of HIV infection was higher among alcohol users (9.5 %) than non-users (1.6 %) (*P* = 0.01). The proportion of HIV infection was higher in women ever had symptom of STI than their counter parts (1.5 %) (*P* = 0.001) (Table 2).

The prevalence of HBV infection was 1 (5 %) and 2 (6.2 %) among women who had history of surgical operation and sharing of sharp objects, respectively. However, the difference was not statistical significant (*P* > 0.05). The mean age of first sexual intercourse was 19.6 year in HBV infected women (Table 3). Tables 2 and 3 depicted the risk factor for HIV and HBV. HIV-HBV co-infection was not found in this study. Furthermore, statistically significant association was not observed for sero-prevalence of HIV and HBV for any of the explanatory variables.

**Table 3:** Association of explanatory variables and HBV infection in young women aged from 15 to 24 years seeking abortion care at Bahir Dar city, 2015

Variables	HBsAg status		*P*-value
	Positive N (%)	Negative N (%)	
History of blood transfusion			
Yes	0	5 (100)	
No	7 (2)	348 (98)	1.00
History of tooth extraction			
Yes	1 (2.3)	412 (97.7)	0.59
No	6 (1.9)	311 (98.1)	
History of genital mutilation			
Yes	4 (2.5)	156 (97.5)	0.7
No	3 (1.5)	197 (98.5)	
History of surgical operation			
Yes	1 (5)	19 (95)	0.33
No	6 (2)	333 (98)	
History of sharing of sharp objects			
Yes	2 (6.2)	30 (93.8)	0.12
No	5 (1.5)	323 (98.5)	
Unprotected sex with older man			
Yes	1 (2)	49 (98)	1.00
No	6 (1.9)	304 (98.1)	
Type of sexual partner			
Student	2 (2.9)	67 (97.1)	0.07
Business man	0	69 (100)	
Drivers	0	48 (100)	
Soldier/police man	0	10 (100)	
Teachers	2 (11.8)	15 (88.2)	
All the above types	0	5 (100)	
Other	3 (2.1)	138 (97.9)	
Alcohol drinking habit			
Yes	2 (4.8)	40 (95.2)	0.19
No	5 (1.6)	313 (98.4)	
Ever had symptom of STI			
Yes	1 (5)	19 (95)	0.33
No	6 (1.8)	334 (98.2)	
Mean age of first sexual intercourse	19.3	18.4	0.31
Mean number of sexual partners	1.3	1.4	0.76

## Discussion

The present study demonstrated the first in its kind for the prevalence of HIV and HBV in young women aged 15–24 years seeking abortion care in Ethiopia. As the study participants were young women seeking abortion care, this study could show the magnitude of sexually acquired HIV and HBV infections risks. Therefore, this study participant deemed good representatives of HIV and HBV prevalence acquired through unprotected sex in the general population.

There are no data on the prevalence of HIV and HBV in young women seeking care for abortion of unwanted pregnancies in Ethiopia therefore we could not compare our results directly with similar study participants.

The prevalence of HIV infection in this study conforms to a report from the study in Cameroon (2.5 %) [22]. However, the prevalence of HIV infection in aborted women was higher than the current national HIV prevalence in young women (0.6 %) and adult (1.3 %) [23]. Likewise, HIV prevalence was higher than studies conducted in Bahir Dar University female students (1.5 %) [24] and Southern Ethiopia (1.8 %) [3]. However, it was lower than the study conducted in Addis Ababa, Bahir Dar, Gondar, Ethiopia [5, 16, 18, 25], Nigeria and Congo [26, 27]. The possible explanation for this difference could be due to difference in risk for HIV acquisition, socio-behavioral and degree of awareness about HIV transmission and prevention as our study participants were different from the above studies in age and purpose of attending the health institutions.

The HBsAg prevalence in this study (1.9 %) is lower than the prevalence of HBsAg in pregnant women in Jimma (3.7 %), Bahir Dar (3.8 %), Gondar (4.7 %), Addis Ababa (3 %), Dessie (4.9 %), Ethiopia [3, 5, 15–17]. Moreover, higher prevalence was reported in Uganda (11.8 %) and Nigeria (16.5 %) [28, 29]. This difference might be attributable to differences in risk for HBV acquisition, the impact of education level and behavioral differences.

The majorities of HIV positive participants in this study were either illiterates or had education of grade 1–8. This was coherent with previous studies in Ethiopia [5, 15] and Mozambique [30]. The possible reason for higher prevalence of HIV infection among illiterate women might be due to lack of awareness about the route of transmission and prevention methods [15].

In this study the prevalence of HIV infection was significantly higher among divorced women compared to married and single (*P* = 0.009). Likewise, similar finding was reported in Kenya [31]. This might be due to transactional relationships with older men, multiple sexual partners and unsafe sexual intercourse after divorce. In addition, marriage at a younger age and the transition from virginity to frequent unprotected sex is likely lead to frequent engagement in sex even after the termination of marriage [32].

The proportion of HBV infection in the present study was significantly higher in the age group of 15–17 years old. This was comparable to a study done in South Africa [10]. This might be due to the fact that young women undergo significant transitions in life style and maturity which will place them at different vulnerabilities at different time points.

In this study, the distribution of HIV and HBV infection were significantly higher among alcohol user women than their counter parts. Alcohol use is one of the common factors that lead young people to exhibit risky sexual behaviours [33–35]. The prevalence of HIV infection was significantly higher among women who had symptom of other STI (*P* = 0.001) which conforms to previous studies conducted in Jimma [3] and Peru [2]. It is a fact that other STI symptoms influence the transmission of HIV and HBV.

In this study, the sero - prevalence of HBV and HIV infection did not show any correlation with history of blood transfusion, surgical operation, sharing of sharp objects and local risk factor like genital mutilation. This is similar to previous studies in HIV infected children and pregnant women in Ethiopia [5, 36]. In the present study, the number of women that had history of blood transfusion or previous surgery was small which might contributed to the absence of association with HBV and HIV infection.

This study has the following main limitations: detection of other markers of HBV like HBeAg and DNA was not possible because of lack of laboratory setup. Interviewing sensitive questions like number of sexual partners to vulnerable groups have a social desirability bias so that potential risk factors for HIV and HBV could not be identified. Moreover, due to the small number of participants it was difficult to explore the risk factors for infections.

## Conclusions

Though there were no co-infections, the prevalence of HIV and HBV infections among young women seeking abortion care is high. New infection among these young women suggested that the disease is not under control yet in the country. Therefore, appropriate HIV and HBV prevention, treatment and care services must be reached to these most risk populations.
